# Editorial

**Published:** 1900-07-15

**Authors:** 


### EDITORIAL.

THE editor of the *International Dental Journal* attended the recent meeting of the American Medical Association at Atlantic City, and the proceedings of the Section of Stomatology evidently interested him. The profound conclusions of these eminent medical specialists and experienced dental practitioners and educationists seems to have convinced Brother Truman that the ideal of dental education must eventually be the degree of Doctor of Medicine. Apparently, however, from his editorial in the July *International Journal*, Brother Truman is not quite ready to sacrifice at once all his professional traditions, and a life experience devoted to practical dental pedagogics, although he seems to believe that dentistry will ultimately be practiced as a specialty of medicine. The following quotation from the editorial will serve to state his views:

"Dental education has been gradually tending toward the medical degree for the past thirty years, and those who have watched the trend of thought have long since come to the conclusion that at no distant period dentistry would be merged into medicine, and that the dentist of the future would practice as a specialist under the one general title of Doctor of Medicine."

We do not wish to question the personal sincerity of this statement, but we are inclined to think it would be exceedingly difficult to formulate a sufficient argument for such a conclusion. We recognize the existence of such a sentiment, but believe it more fictitious than real. Is it possible to produce any considerable statistical or even favorable sentimental evidence that will justify such a statement? For example, do we find that relatively a larger proportionate number of dental graduates are seeking the medical degree in addition, or that there is any considerable increase in the number of medical graduates seeking dental qualifications? We acknowledge an existing sentiment that more knowledge of medical science and practice would be of great benefit to the dental practitioner, but we do not find dental practitioners in any considerable numbers in attendance at post-graduate medical schools, with the intent of further enlarging their equipment for dental practice.

Does the statement find any justification in the system of dental education? A comparison of the curriculum of to-day with that of thirty years ago will show that little has been added of medical technique, while great additions have been made along the line of the medical sciences, scarcely anything in the way of clinical or practical medicine. It may be that clinical or technical dentistry has made greater advances than similar departments of medicine.

We do not see any prospect of dentistry becoming a specialty of medicine in the very near future, but we do see indications that at no very distant date the whole system of medical education, if not its practice, will be put on a different basis. In fact, educationally, there is a division in the sources as well as methods of training men for the practice of medicine in specialties. In the largest and most progressive schools there are practically two faculties, though nominally but one. The fundamental or scientific chairs are filled by scientific men, who devote their entire time to work in their sciences and imparting instruction. These men may have medical degrees, but they are not, as a rule, practitioners. On the other hand, the clinical teachers hold their positions not primarily because they are scientists, but because of their pre-eminent qualifications as successful practitioners. And we predict that the time will come when the degree of Medical Science will be the foundation upon which will be founded a medical, a surgical, a dental and probably many other well-defined specialties, each designated by its own appropriate and significant title. In this way dentistry may become a specialty of medical science, but hardly on the present basis of medical education.

It seems rather unreasonable to ask a student who is qualifying to practice dentistry to master the two years of almost purely technical medical work in a medical school, when he will be compelled to spend a like amount of time in another course to qualify for dental practice, thus necessitating six years of preparation, and, as Prof. Truman states in another part of the same editorial, much of the work done in the medical course has actually disqualified the student for dental technics. Why then should a student's chances of becoming a successful practitioner of dentistry be jeopardized for the sake perhaps of being associated with an older and possibly more respected profession?

We can not see that there has been any particular conveying of the lines of dental and medical practice, although there may be from the educational standpoint. They seem to have moved forward together, side by side, both dependent on and advancing only because of results secured from researches in the general sciences upon which both are founded. If future advances are to be made on the same basis is it not more becoming for the dental profession to remain loyal to its past, magnifying the significance of its degree, by demonstrating its usefulness as an important branch of the healing art based upon the principles established by medical science?

